# Association study of OPRM1 polymorphisms with Schizophrenia in Han Chinese population

**DOI:** 10.1186/1471-244X-13-107

**Published:** 2013-04-05

**Authors:** Saidan Ding, Bicheng Chen, Yong Zheng, Qin Lu, Leping Liu, Qǐ -Chuan Zhuge

**Affiliations:** 1 Zhejiang Provincial Key Laboratory of Aging and Neurological Disease Research, Department of Surgery, the First Affiliated Hospital of Wenzhou Medical College, Wenzhou, Zhejiang, China; 2 Clinical Laboratory, the Fifth People’s Hospital of Ruian city, Wenzhou, Zhejiang, China; 3 Neurosurgery Department, the First Affiliated Hospital of Wenzhou Medical College, Wenzhou, Zhejiang, China

**Keywords:** Schizophrenia, OPRM1 gene, Polymorphisms

## Abstract

**Background:**

The expression of μ-opioid receptor has important role in cognitive dysfunction in Schizophrenia (SZ). The results of studies about the association of polymorphisms of μ-opioid receptor gene (OPRM1) with SZ were inconsistent.

**Methods:**

We conducted a case–control study to investigate the genetic association between OPRM1 polymorphisms and SZ among the Han chinese population. 264 SZ patients and 264 age-matched control subjects were recruited. Four SNPs of OPRM1 were successfully genotyped by using PCR-RFLP.

**Results:**

Of four polymorphisms, rs1799971 and rs2075572 were shown to associate with SZ. Compared with the A allele of rs1799971 and C allele of rs2075572, the G allele of rs1799971 and rs2075572 was associated with an almost 0.46-fold risk (OR = 0.46, 95% CI: 0.357-0.59, P < 0.01) and 0.7-fold risk (OR = 0.707, 95% CI: 0.534-0.937, P = 0.015) of the occurrence of SZ,. When subjects were divided by gender, rs1799971 remained significant difference only in males (OR = 0.309, 95% CI: 0.218-0.439 for G allele, P < 0.01), and rs2075572 only in females (OR = 0.399, 95% CI: 0.246-0.648 for G allele, P < 0.01). In secondary analysis with subsets of patients, the G allele of rs1799971 (compared to the A allele) was associated with a decreased risk of all patients and male patients with apathy symptoms (OR = 0.086, 95% CI: 0.048-0.151, P = 0.01; OR = 0.083, 95% CI: 0.045-0.153, P < 0.01), and the G allele of rs2075572 (compared to the C allele) was associated with a decreased risk of all patients and female patients with positive family history (OR = 0.468, 95% CI: 0.309-0.71, P < 0.01; OR = 0.34, 95% CI: 0.195-0.593, P < 0.01). In addition, haplotype analysis revealed that two SNP haplotypes (A-C-C-G and G-C-C-A) were associated with decreased risks of SZ (P < 0.01). The other two (G-C-C-G and G-G-C-G) with increased risks of SZ (P < 0.01).

**Conclusions:**

The present study demonstrated for the first time that the OPRM1 polymorphism may be a risk factor for schizophrenia in the Han Chinese. Further studies are needed to give a global view of this polymorphism in pathogenesis of schizophrenia in a large-scale sample, family-based association design or well-defined subgroups of schizophrenia.

## Background

Schizophrenia (SZ) is a common, chronic and complex psychiatric disorder, affecting 1.0% of the worldwide population [1]. The disorder presents delusions and hallucinations, reduced interest and drive, altered emotional reactivity and disorganized behavior [2]. The data collected from family, twin, and adoption studies show unequivocally that SZ is a predominantly genetic disorder, and heritability estimates for SZ range from 70% to 80% [3]. Traditionally, Genetic research of SZ focused on identifying linkage regions or on candidate genes and polymorphisms. Now a few studies have obtained positive replications, including chromosomal locus and susceptibility genes [4,5]. Apparently, however, effect sizes are small and individual studies were not replicated by other groups. Recently, genome-wide association studies (GWAS) provided new evidence for SZ genetics, by investigating single nucleotide polymorphism (SNP) and copy number variation [6-9]. Some results suggest that multiple functional variants from genes in neurodevelopmental pathways contribute to development of SZ [8], while the biological mechanism is undefined.

Theμ-opioid receptor (MOR; encoded by genetic locus OPRM1) is widely distributed in the brain, and is highly expressed in the thalamus, caudate putamen and globus pallidus [10]. MOR has a high affinity for b-endorphin and enkephalin but a low affinity for dynorphin (which preferentially binds to the k-opioid receptor), and it also binds to exogenous opioid drugs (e.g. morphine, heroin and methadone) with a high affinity. MORs are thought to be responsible for most opioidergic actions such as euphoria, analgesia and opiate drug withdrawal [11]. Disruption of the MOR gene (Oprm) in mice abolishes morphine-induced analgesia, place-preference activity and physical-dependence, even in the presence of intact d- and k-opioid receptors (DOR and KOR) [12,13]. More than 250 SNPs have been identified in the OPRM1 gene [14-16]. The A118G SNP in exon1 leads to an amino acid substitution that changes the putative N-glycosylation site [17]. A118G (rs1799971) has been studied in relation to tardive dyskinesia in selected groups of patients in Japan and China [18,19]. This polymorphism, leading to a substitution of asparagine (Asn) for aspartic acid (Asp) at amino acid position 40, reduces through changed receptor glycosylation the affinity for endogenous opioids [20].

In addition, intronic sequence can be involved in alternative DNA splicing. To date, nine human OPRM1 splice variants have been identified [21-23]. They contain the same exons 1, 2 and 3 as the original human OPRM1,which normally has four exons. However, they differ from this sequence and from each other by splicing downstream from exon3. All splice variants result in amino acid sequence changes in the C-terminus of the MOR and may affect the activity (e.g. phosphorylation and internalization) of the receptor. rs2075572 (C/G) is in intron2; rs648893 (C/T) is in intron3; rs671531 (A/G) is in the downstream region of the OPRM1 gene.

SZ has been linked to dysfunction of prefrontal cortical (PFC) g-aminobutyric acid (GABA) neurons and appears neurodevelopmental in nature [24-28]. Opioids suppress GABA neuron activity [29], so higher MOR mRNA levels existing in SZ may contribute to suppressed PFC GABA neuron activity [30-32]. The rs1799971 polymorphism of the OPRM1 gene has an impact on μ-opioid receptors functioning [18,33]. So the G allele rs1799971 leads to decreased expression of the receptor [34]. There is also a possible relationship between dysfunction of this receptor and opiates abuse in SZ patients [35].

**Figure 1:**
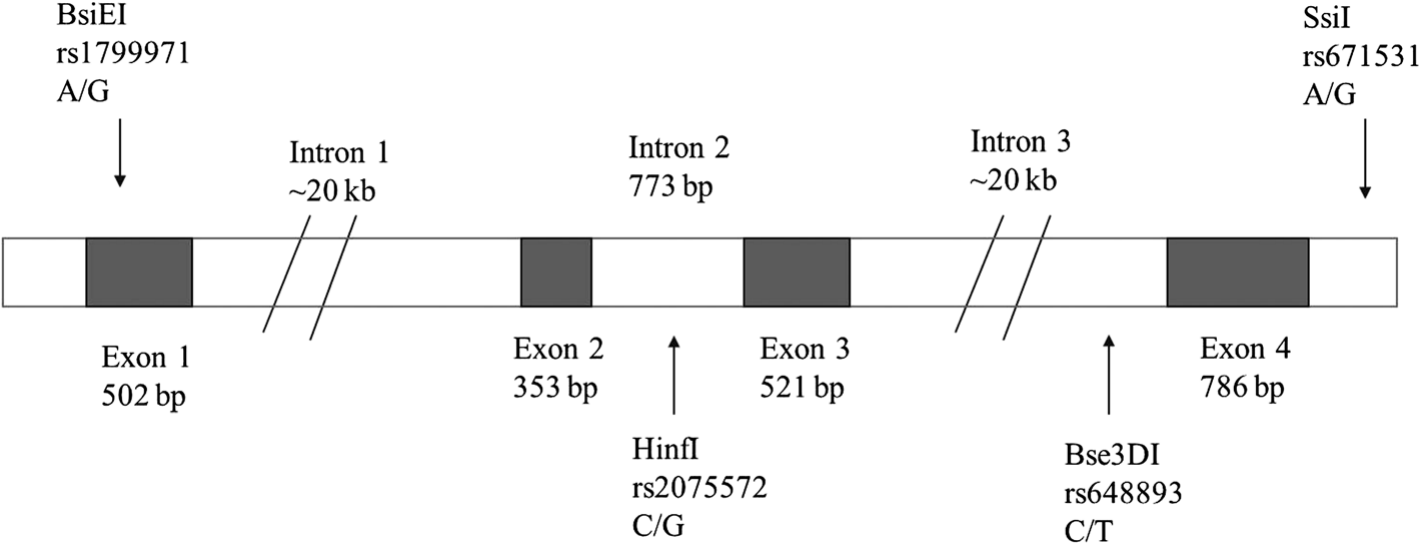
The human μ-opioid receptor gene (OPRM1) structure and 4 SNP variants.

Therefore, we designed an association study aimed at OPRM1 gene polymorphism in relation to SZ-----a case–control study to investigate the association of alleles, genotypes, haplotypes of 4 SNPs (rs1799971, 118A/G or Asn40Asp; rs2075572, C/G; rs648893, C/T; rs671531, A/G) of OPRM1 (Figure 1) with SZ in a group of the Han Chinese population.

## Methods

### Subjects characteristics

The studied sample consisted of 264 patients with SZ (154 males and 110 females; mean age: 37.44 ± 11.04 years old) and 264 healthy controls (146 males and 118 females; mean age: 36.38 ± 12.27 years old). All patients were recruited from inpatient or outpatient in the Fifth People’s Hospital of Ruian city. These patients were unrelated Han Chinese born and living in southern Zhejiang province, and all their biological grandparents were of Han Chinese ancestry. The consensus diagnoses were made by at least two experienced psychiatrists according to the Diagnostic and Statistical Manual of Mental Disorders-Fourth Edition IV (DSM-IV) (1994) diagnostic criteria for paranoid SZ. Individuals with a history of severe medical complications, such as diabetes, cardiovascular disease, hypertension, neurological diseases, any concomitant major psychiatric disorders, or substance dependence were excluded.

Healthy controls were recruited from hospital staffs who had volunteered to paticipate.

The Mini-International Neuropsychiatric Interview was performed in the control group [36] followed by an interview with a psychiatrist. All individuals with mental illnesses (addictions, schizophrenia, mood disorders) were excluded from the control group. All participants signed informed consent forms to join the study participation, and these forms remain stored.

Those individuals with personal or family history of neurological diseases and mental health, such as diagnoses of major depressive disorder, SZ or bipolar mood disorder were excluded. The controls living in the same area as patients were well matched to patient group on gender, age (F = 0.699, p = 0.403) and ethnicity.

The approval of Clinical research professional committee of the First Affiliated Hospital of Wenzhou Medical College was obtained, and all subjects, both cases and controls, gave written informed consent to participate.

### Selection of subsets

The psychiatric symptoms of the patients were rated using the Brief Psychiatric Rating Scale (BPRS) [37,38]. The principal component analyses of the symptoms assessed by the BPRS were also performed, and five factors emerged: Delusion of reference, genuine auditory hallucination, delusion of persecution, bizarre behavior, and apathy delusion [39]. The patients were classified into five subgroups according to clinical symptoms. All patients have more than one symptom.

The second subset was composed of patients stratified by age-of-onset, divided into two subgroups (age 18 or less and more than 18; 18 years and >18 year). Referring to our previous studies, the age at onset of schizophrenia was defined as the age when positive symptoms (either delusions or hallucinations) first became apparent based on interviews and supplemental clinical information obtained from medical records and family informants [40,41]. Onset before 19 years of age is known as early age-of-onset [42]. The third subset was composed of patients stratified by family history, divided into two subgroups (positive and negative family history). The definition of positive family history of SZ was verified presence of one or more, 1st or 2nd degree relatives having SZ [43].

### Molecular analysis

Blood samples were collected with the anticoagulant EDTA K2 and stored at −20°C. Genomic DNA was extracted from the blood using a DNA Extraction Kit (TaKaRa Bio Group, Japan). OPRM1 polymorphisms were genotyped by PCR-RFLP and the condition was displayed in the Table 1. We selected the four single nucleotide polymorphisms (SNPs) in the OPRM1 gene according to the dbSNP http://www.ncbi.nlm.nih.gov/SNP/ and the international HapMap project http://www.hapmap.org/, including rs1799971, rs2075572, rs648893, rs671531.

**Table 1:** PCR-RFLP conditions for OPRM1 gene polymorphisms

**SNP**	**Primer**	**Base change**	**Annealing temperature**	**Restriction enzyme**
rs1799971	5’GTCTCGGTGCTCCTGGCTACCTCGC3’(F)	A/G	65	BsiEI
	5’TTCGGACCGCATGGGTCGGACCGGT3’(R)			
rs2075572	5’TAAGTTAGCTCTGGTCAAGGCTAAGAAT3’(F)	C/G	55	HinfI
	5’ATCATCAGTCCATAGCACACGGTAA3’(R)			
rs648893	5’AACAGATTAGGTCATTCTCACTTTA3’(F)	C/T	50	Bse3DI
	5’GCTTTAGCATAATAGTGCCAGTTCC3’(R)			
rs671531	5’ATCTGGCTAAGGCATCATTTTCACC3’(F)	A/G	53	SsiI
	5’TTTCACATCCAAGTAACTACACAGG3’(R)			

These markers cover 80045 bp of the OPRM1 coding region (Figure 1).

The four SNPs were analyzed by polymerase chain reaction-restriction fragment length polymorphism (PCR-RFLP) analysis. The information of primers and PCR-RFLP analysis is given in Table 1. The PCR amplification was performed in a 50 μl volume containing GC Buffer (TaKaRa), 200 mM of each dNTPs, 0.3 mM of each primer, 1 U of Taq DNA polymerase, and 40 ng of the genomic DNA. The conditions used for PCR amplification were an initial denaturation phase at 94°C for 5 min, followed by 36 cycles at 94°C for 30 sec, annealing at 50-65°C for 30–60 sec, and extension at 72°C for 30 sec, followed by a final extension phase at 72°C for 7 min. A 10 μL aliquot of the PCR product mixtures was completely digested with 1–3 units of restriction enzyme in 2–4 hours at 37°C. Digestion products were visualized through ethidium bromide staining after electrophoresis in 1%-3% agarose gels.

### Power calculations

Power calculations were performed using the Genetic Power Calculator [44]. We assumed that the polymorphism and the disease locus were in complete linkage disequilibrium (LD) and that they had the same allele frequencies, ie, the polymorphism was the disease locus. Assuming a recessive disease locus and a disease prevalence of 0.02. Our exploratory sample size of 264 cases and 264 control subjects had an 80% power to detect a susceptibility locus with a genotypic relative risk ≥ 3 at ≤ 0.10 for SNPs with minor allele frequencies ≥ 0.2. A dominant model had 80% power to detect a locus with a genotypic relative risk ≥ 1.7 and a SNP minor allele frequency ≥ 0.1 atα ≤ 0.1.

### Statistical analysis

Genotype and allele frequencies were compared between patients and controls using the SHEsis software [45]. HWE was also tested using this program. The standardized measure of linkage disequilibrium (LD) coefficients (D’), haplotype frequency, haplotype block, and haplotype association were also assessed using the SHEsis software. Single-SNP analysis was carried out using the Pearson chi-square test on allele and genotype counts. Unconditional logistic regression analysis models were used to evaluate the relationships between different genotypes and disease risk [Odds ratios (OR), 95% confidence intervals (95% CI)] adjusted by age, a p-value of less than 0.05 was considered as statistically significant. Correction for multiple testing of SNPs that are in LD with each other was applied according to the method introduced by Li & Ji [46] which improves an approach proposed by Nyholt [47], and consequently single-test p-values < 0.01 were considered to be significant.

## Results

### Comparison of the allelic and genotypic frequencies of SNPs in patient and control group

Four OPRM1 SNPs were detected in 528 ethnic Chinese schizophrenic patients. These were rs1799971 (118A/G or Asn40Asp), rs2075572 (C/G), rs648893 (C/T) and rs671531 (A/G). Among the four SNPs, only rs1799971 was in coding region, causing an amino acid substitution (Asn40 to Asp40) that removes a highly conserved N-glycosylation site in the extracellular domain of the protein. A significant deviation from the Hardy-Weinberg equilibrium was not found in either the patient group or in the control group for all four SNPs. Genotypic and allelic frequencies of four SNPs are shown in Table 2. Statistical analysis revealed a significant difference for the rs1799971, rs2075572 polymorphisms, while other polymorphisms did not show any significant differences.

**Table 2:** Distribution of OPRM1 genotypes and alleles between SZ cases and controls in all, female and male samples*

**SNP**	**Haplotype/Allele**	**All**				**Male**				**Female**			
		**Controls**	**Cases**	**OR (95%)**	**P value**	**Controls**	**Cases**	**OR (95%)**	**P value**	**Controls**	**Cases**	**OR (95%)**	**P value**
rs799971	AA	68	132	1.00(referent)		31	93	1.00(referent)		37	39	1.00(referent)	
	AG	133	102	**0.417(0.281-0.618)**	<0.00001	85	50	**0.208(0.121-0.358)**	<0.00001	48	52	1.063(0.582-1.941)	0.84
	GG	63	30	**0.257(0.152-0.436)**	<0.00001	30	11	**0.127(0.057-0286)**	<0.0.00001	33	19	0.564(0.273-1.164)	0.12
	A	269	366	1.00(referent)		147	236	1.00(referent)		122	130	1.00(referent)	
	G	259	162	**0.46(0.357-0.59)**	<0.00001	145	72	**0.309(0.218-0.439)**	<0.00001	114	90	0.741(0.511-1.073)	0.11
	HWE(P)	0.9	0.14			0.4	1.00			0.37	0.68		
rs2075572	CC	140	166	1.00(referent)		79	84	1.00(referent)		61	82	1.00(referent)	
	CG	100	82	0.697(0.481-1.010)	0.06	51	55	1.053(0.643-1.725)	0.84	49	27	**0.383(0.213-0.689)**	0
	GG	24	16	0.594(0.301-1.171)	0.59	16	15	0.951(0.437-2.071)	0.9	8	1	**0.087(0.01-0.723)**	0.02
	C	380	414	1.00(referent)		209	223	1.00(referent)		171	191	1.00(referent)	
	G	148	114	**0.707(0.534-0.937)**	0.02	83	85	0.96(0.672-1.371)	0.82	65	29	**0.399(0.246-0.648)**	<0.00001
	HWE(P)	0.32	0.68			0.96	0.91			0.56	0.68		
rs648893	CC	107	116	1.00(referent)		55	69	1.00(referent)		52	47	1.00(referent)	
	CT	113	110	0.888(0.61-1.292)	0.54	59	57	0.755(0.453-1.259)	0.28	54	53	1.102(0.631-1.925)	0.73
	TT	44	38	0.76(0.455-1.268)	0.29	32	28	0.671(0.36-1.253)	0.21	12	10	0.848(0.331-2.171)	0.73
	C	327	342	1.00(referent)		169	195	1.00(referent)		158	147	1.00(referent)	
	T	201	186	0.885(0.689-1.137)	0.34	123	113	0.796(0.573-1.105)		78	73	1.006(0.681-1.486)	0.98
	HWE(P)	0.13	0.06			0.96	0.84			0.38	0.21		
rs671531	AA	102	109	1.00(referent)		56	68	1.00(referent)		46	41	1.00(referent)	
	AG	116	122	0.968(0.665-1.408)	0.87	63	69	0.893(0.544-1.465)	0.65	53	53	1.091(0.613-1.942)	0.77
	GG	46	33	0.628(0.37-1.065)	0.09	27	17	0.482(0.237-0.981)	0.04	19	16	0.902(0.406-2.004)	0.8
	A	320	340	1.00(referent)		175	205	1.00(referent)		145	135	1.00(referent)	
	G	208	188	0.851(0.663-1.092)	0.2	117	103	0.752(0.539-1.048)	0.09	91	85	1.003(0.688-1.463)	0.99
	HWE(P)	0.2	0.08			0.95	1.00			0.67	1.00		

*According to the method of multiplicity correction for SNPs in LD that was introduced by Li & Ji [54], we considered p-values < 0.01 as significant.

A significant difference in both genotype and allele at rs1799971 was found between SZ patients and controls. Compared with rs1799971 AA, subjects with AG and GG genotypes were associated with a decreased risk (OR = 0.417, 95% CI: 0.281-0.618, P < 0.00001; OR = 0.257, 95% CI: 0.152-0.436, P < 0.00001). The frequency of the A allele of rs1799971 were also lower in patients (50.9%) than in controls (69.3%, OR = 0.46, 95% CI: 0.357-0.591, P < 0.00001). There was a significant difference in allelic frequencies at rs2075572. Compared with rs2075572 C allele, subjects with G allele were associated with a decreased risk (OR = 0.707, 95% CI: 0.534-0.937, P = 0.015).

To determine the gender effect, genotype and allele frequency in both sexes were assessed (Table 3). In male samples, rs1799971 showed significant difference between SZ patients and controls in the genotype and allele. Compared with AA, subjects with AG and GG genotypes were associated with a decreased risk (OR = 0.208, 95% CI: 0.121-0.358, P < 0.00001; OR = 0.127, 95% CI: 0.057-0.286, P < 0.00001). Compared with A allele, subjects with G allele were associated with a decreased risk (OR = 0.309, 95% CI: 0.218-0.439, P < 0.00001). In female samples, there was a significant difference in both genotypic and allelic frequencies at rs2075572 between SZ patients and controls. Compared with CC genotype, subjects with CG and GG genotypes were associated with a decreased risk (OR = 0.383, 95% CI: 0.213-0.689, P = 0.001; OR = 0.087, 95% CI: 0.01-0.723, P = 0.024). Compared with C allele, subjects with G variant allele were associated with a decreased risk (OR = 0.399, 95% CI: 0.246-0.648, P < 0.00001).

**Table 3:** Comparison of the genotypic and allelic frequencies of rs1799971 and rs2075572 in different kinds of patients compared with controls by BsmI polymorphisms*

**SNP**	**Gender**	**Haplotype**	**Controls**	**Clinical symptoms**									
				**Delusion of reference**	**OR(95% CI)**	**Genuine auditory hallucination**	**OR(95% CI)**	**Delusion of persecution**	**OR(95% CI)**	**Bizarre behavior)**	**OR(95% CI)**	**Apathy**	**OR(95% CI)**
rs799971	All	AA	68	53	1.00(referent)	40	1.00(referent)	34	1.00(referent)	51	1.00(referent)	30	1.00(referent)
		AG	133	92	0.888(0.568-1.387)	78	0.997(0.617-1.612)	69	1.038(0.627-1.718)	101	1.013(0.648-1.581)	57	0.971(0.572-1.65)
		GG	63	29	0.591(0.335-1.042)	21	0.405(0.204-0.803)	19	0.603(0.312,1.164)	29	0.614(0.347-1.086)	20	0.72(0.371-1.394)
		A	269	198	1.00(referent)	158	1.00(referent)	137	1.00(referent)	203	1.00(referent)	117	1.00(referent)
		G	259	150	0.787(0.599-1.033)	120	0.789(0.589-1.057)	107	0.811(0.598-1.101)	159	0.813(0.622-1.064)	97	0.861(0.626-1.184)
	Male	AA	31	26	1.00(referent)	24	1.00(referent)	16	1.00(referent)	21	1.00(referent)	15	1.00(referent)
		AG	85	45	0.631(0.335-1.19)	48	0.729(0.385-1.383)	32	0.729(0.352-1.51)	44	0.764(0.394-1.483)	46	1.118(0.548-2.282)
		GG	30	11	0.437(0.184-1.039)	10	0.431(0.176-1.051)	7	0.452(0.163-1.254)	11	0.541(0.223-1.312)	5	0.344(0.111-1.066)
		A	147	97	1.00(referent)	96	1.00(refrent)	64	1.00(referent)	86	1.00(referent)	76	1.00(referent)
		G	145	67	0.7(0.476-1.031)	68	0.718(0.488-1.057)	46	0.729(0.468-1.135)	66	0.778(0.524-1.154)	56	0.747(0.494-1.131)
rs2075572	All	CC	140	91	1.00(referent)	83	1.00(referent)	68	1.00(referent)	90	1.00(referent)	76	1.00(referent)
		CG	100	69	1.062(0.708-1.591)	49	0.827(0.534-1.279)	43	0.885(0.559-1.402)	78	1.213(0.816-1.805)	29	**0.534(0.324-0.88)**
		GG	24	14	0.897(0.441-1.825)	7	0.492(0.203-1.192)	11	0.944(0.437-2.038)	13	0.843(0.408-1.74)	2	**0.154(0.035-0.667)**
		C	380	251	1.00(referent)	215	1.00(referent)	179	1.00(referent)	258	1.00(referent)	181	1.00(referent)
		G	148	97	0.992(0.734-1.342)	63	0.752(0.536-1.056)	65	0.932(0.663-1.312)	104	1.035(0.769-1.392)	33	**0.468(0.309-0.71)**
	Female	CC	61	35	1.00(referent)	45	1.00(referent)	31	1.00(referent)	25	1.00(referent)	64	1.00(referent)
		CG	49	20	0.711(0.366-1.384)	27	0.747(0.407-1.371)	24	0.854(0.45-1.621)	27	1.344(0.694-2.605)	19	0.37(0.196-0.698)
		GG	8	1	0.218(0.026-1.815)	1	0.169(0.02-1.404)	1	0.246(0.029-2.056)	0	-	0	-
		C	171	90	1.00(referent)	117	1.00(referent)	86	1.00(referent)	77	1.00(referent)	147	1.00(referent)
		G	65	22	0.643(0.372-1.111)	29	0.652(0.397-1.072)	26	0.795(0.471-1.342)	27	0.922(0.547-1.557)	19	0.34(0.195-0.593)

*According to the method of multiplicity correction for SNPs in LD that was introduced by Li & Ji [54], we considered p-values < 0.01 as significant.

### Secondary analysis with assumedly genetic subsets of patients

When stratifying patients according to five main clinical symptoms (delusion of reference, genuine auditory hallucination, delusion of persecution, bizarre behavior, and apathy), the genotypic and allelic frequencies at rs1799971 and rs2075572 sites were compared between every subgroup and the original control group (Table 3). Only in the subgroup**(s)** with apathy symptoms, the frequency of the G allele of rs2075572 was 15.42% SZ with apathy symptoms, significantly lower than that in controls (28.03%). The G allele of rs2075572 (compared to the A allele) was associated with a decreased risk of all patients and female patients with apathy symptoms (OR = 0.468, 95% CI: 0.309-0.71, P < 0.00001; OR = 0.34, 95% CI: 0.195-0.593, P < 0.00001).

When stratifying patients by age-of-onset, the genotypic and allelic frequencies of rs1799971 and rs2075572 in two subgroups were compared with that of the original control group. However, there was no significant association of the minor allele of the two SNP with a risk of SZ with early age-of-onset (age 18 or less).

When stratifying patients by family history, the G allele of rs1799971 in all and male patients with positive family history (7.61%, 7.56%) was lower frequently than that of controls (49.05%; 49.66%), and the OR values were 0.086 and 0.083, respectively (95% CI: 0.048-0.151, P =0.01; 95% CI: 0.045-0.153, P < 0.00001). While the OR value for the G allele of rs2075572 was 0.499 and 0.347 (versus the C allele) among all and female patients of negative family history (95% CI: 0.354-0.704, P < 0.00001; 95% CI: 0.203-0.595, P < 0.00001).

### LD mapping result and LD relationships among the SNPs

**Figure 2:**
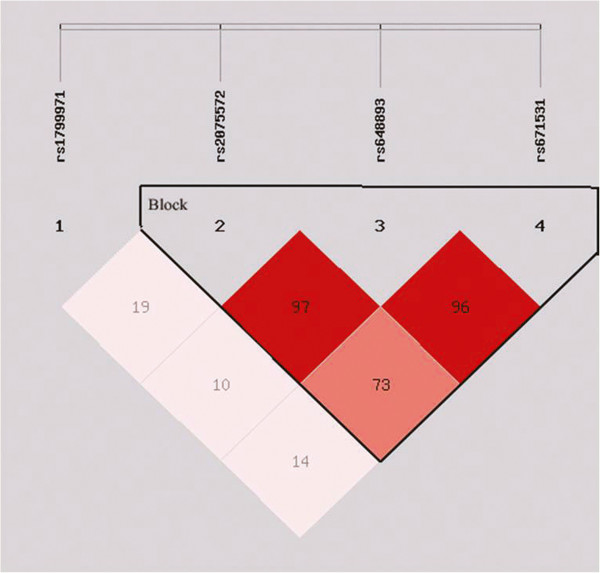
**Linkage disequilibrium (LD) plot of 4 OPRM1 SNPs in SZ cases and controls.** There was only one LD block in OPRM1 gene. (The numbers in the squares are D’X 100. Squares are colored bright red if the D’value is high (i.e. LD is strong) and the confidence in the value of D’is high as well).

The results of LD between each pair of SNPs are shown in Table 4. The LD analysis revealed that three SNPs (rs2075572, rs648893 and rs671531) were in an LD block (|D’| > 0.9) (Figure 2), which is consistent with other studies [48]. The rs2075572 showed significant LD with rs648893 and rs671531. In addition, there was strong LD between rs648893 and rs671531.

**Table 4:** Linkage disequilibrium test between four OPRM1 gene polymorphisms

**SNP1**	**SNP2**	**Physical distance(bp)**	**r**^**2**^
rs1799971	rs2075572	51207	0.02
rs1799971	rs648893	77832	0
rs1799971	rs671531	79945	0.02
rs2075572	rs648893	26625	0.18
rs2075572	rs671531	28738	0.3
rs648893	rs671531	2113	0.32

The distributions of haplotypes of the four SNPs are listed in Table 5. Haplotypes with four loci (rs1799971, rs2075572, rs648893 and rs671531) of OPRM1 gene polymorphisms were analyzed. We found that the frequencies of haplotypes ‘A-C-C-G and G-C-C-A’ were 2.7% and 4.4% of cases, significantly lower than in controls (15.6% and 10.6%), and the odds ratios were 0.149 (95% CI: 0.084–0.266, P < 0.00001) and 0.386 (95% CI: 0.233–0.638, P < 0.00001) respectively, while the haplotype ‘G-C-C-G and G-G-C-G’ was more frequent in cases than in controls (13% vs. 3.4% and 13.7% vs. 5.1%, P < 0.00001), while the odds ratio values were 4.317 (95% CI: 2.526–7.377, P < 0.00001) and 2.984 (95% CI: 1.881–4.732, P < 0.00001).

**Table 5:** Comparison of haplotype frequencies for four OPRM1 polymorphisms between SZ cases and controls

**Haplotypes**							
**rs1799971**	**rs2075572**	**rs648893**	**rs671531**	**Case (freq)**	**Control (freq)**	**Odds Ratio (95% CI)**	P value*
A	C	C	A	78.74(0.149)	71.94(0.136)	1.119(0.791-1.583)	0.53
A	C	C	G	14.23(0.027)	82.39(0.156)	0.149(0.084-0.266)	<0.01
A	C	T	A	127.67(0.242)	126.27(0.239)	1.022(0.769-1.358)	0.88
A	G	C	A	1.48(0.003)	24.66(0.047)	-	-
A	G	C	G	46.88(0.089)	60.74(0.115)	0.753(0.503-1126)	0.17
G	C	C	A	22.97(0.044)	55.75(0.106)	0.386(0.233-0.638)	<0.01
G	C	C	G	68.80(0.130)	17.92(0.034)	4.317(2.526-7.377)	<0.01
G	C	T	A	67.55(0.128)	59.73(0.113)	1.158(0.798-1.681)	0.44
G	G	C	A	21.59(0.041)	1.65(0.003)	-	-
G	G	C	G	72.30(0.137)	26.95(0.051)	2.984(1.881-4.732)	<0.01
G	C	T	G	0.04(0.000)	0.00(0.000)	-	-
G	G	T	G	5.75(0.011)	0.00(0.000)	-	-

## Discussion

The m-opioid receptor MOR; encoded by genetic locus OPRM1) is widely distributed in the brain, and is highly expressed in the thalamus, caudate putamen and globus pallidus [10,11]. MORs are thought to be responsible for most opioidergic actions such as euphoria, analgesia and opiate drug withdrawal [11]. MOR binds to exogenous opioid drugs (e.g. morphine, heroin and methadone) with a high affinity. Clinical effects of some opioid agonists (morphine, methadone) are accompanied by modulation of the dopaminergic, glutamatergic, and GABAergic transmission in the human brain. The evidence of an indirect effect includes increased dopamine release in individuals with SZ [49,50]. Therefore, the mutation of OPRM1gene, leading to the inactivation of MOR and reduced dopamine release, has no susceptibility to SZ.

This study has brought out a finding of an association of the μ-opioid receptor gene (OPRM1gene) polymorphism with SZ again by more SNPs (rs1799971, rs2075572, rs648893 and rs671531). A currently published article has shown that morphine, which acts as a μ- opioid agonist, increases prepulse inhibition of startle reaction that is significantly deficient in patients with SZ.

P < 0.00001 A group of Czech authors found that the rs1799971 polymorphism of the OPRM1 gene was associated with increased risk of SZ in the male population [51]. However, our results show that the probability of having SZ for those with the G variant of rs1799971 and rs2075572 was decreased, compared to people carrying the A-allele and C-allele, suggesting that the “G” allele of rs1799971 and rs207557 might have a protective effect against SZ. A group of Japanese authors tried to document the association between the rs1799971 polymorphism of the OPRM1 gene and tardive dyskinesia in SZ patients. They documented that the G allele was significantly less represented in patients with tardive dyskinesia, which presented its “protective” role [18]. A following Chinese study observed a similar trend of the G allele being less frequent in subjects with tardive dyskinesia in SZ patients [33].

The level of MOR mRNA in SZ was elevated, having important role in cognitive dysfunction [32]. And the G allele of rs1799971 has impact on μ-opioid receptors functioning, leads to decreased expression of the receptor [18,33,34]. Therefore, our results were consistent with the role of μ-opioid receptors in SZ.

In our study a lower frequency of the G allele and the GG genotype in rs1799971 and rs2075572 was found in patients with SZ. The results allow a hypothesis that a possible increased expression of the μ-opioid receptor in individuals with SZ, caused by the absence of the G allele, and may thus lead to the hyperactivity in the dopamine system. There are several possible explanations for the above discrepancies between the previous reports and our results. The first possibility relates to the different ethnicities of the subjects. Some inconsistent results in association studies may be attributed to genetic heterogeneity since the various studies were carried out in distinct ethnic populations. The second possibility relates to the gender effect. When our subjects were divided by gender, one SNPs (rs2075572) was associated with SZ in females, but another SNPs (rs1799971) was associated with SZ in males.

Many studies have demonstrated that there is a gender difference in clinical factors of SZ such as symptomatology, premorbid functioning and age of onset [52]. The gender effect on SZ at genetic level was also reported [53,54].

It is well known that the incidence of complicated diseases like SZ depend on the interaction of multiple factors. Usually no single gene is uniquely responsible for these diseases and environmental factors also play a role in their occurrence. The methods used in individual studies, may have limited power to detect a small effect, or small interactions with other relevant polymorphisms. Limited sample size, etiological heterogeneity and clinical heterogeneity may result in inconsistent results showing different associations between the OPRM1 gene and SZ [18,33,51], and therefore, the conclusion of this study may be viewed in this context. Further investigations using larger sample sizes and family-based studies will undoubtedly add further valuable insight into the implications of the relationship between OPRM1 gene polymorphisms and SZ.

## Conclusion

In conclusion, the present study suggests that OPRM1 gene polymorphisms may be associated with SZ risk in the southern Chinese Han population, and G alleles of rs1799971 and rs2075572 are protective factors in male and female SZ patients respectively, and these associations may largely depend on population characteristics and geographic location.

## Competing interests

The authors’ declare that they have no competing interests.

## Authors’ contributions

Saidan Ding, Leping Liu, Yong Liang, Weilong Hong, Jieya Xie performed laboratory assays. Saidan Ding performed the data-analysis and drafted the manuscript. Jiancheng Wen performed sample collection. Saidan Ding and Bicheng Chen participated in the design of the study and performed phenotypic diagnosis. Qǐ -Chuan Zhuge participated in the design of the study, interpretation of the data, and drafting of the manuscript. All authors read and approved the final manuscript.

## Pre-publication history

The pre-publication history for this paper can be accessed here:

http://www.biomedcentral.com/1471-244X/13/107/prepub
